# Do We Need Systematic Reviews of Research Priority Setting? A Proposal for a New Concept on Conducting Systematic Reviews of Research Priority Setting Exercises

**DOI:** 10.1002/cesm.70079

**Published:** 2026-03-31

**Authors:** Mona Nasser, Sumanth Kumbargere Nagraj, Seilin Uhm, Prashanti Eachempati, Soumyadeep Bhaumik

**Affiliations:** ^1^ Peninsula Dental School, Faculty of Health University of Plymouth Plymouth Devon UK; ^2^ School of Health Sciences University of Southampton Southampton Hampshire UK; ^3^ Meta‐research and Evidence Synthesis Unit, The George Institute for Global Health University of New South Wales Sydney Australia; ^4^ Meta‐research and Evidence Synthesis Unit The George Institute for Global Health New Delhi India; ^5^ Department of Public Health Water Sisulu University Mthatha Republic of South Africa

**Keywords:** evidence synthesis, research agenda, research priority setting, stakeholder engagement, systematic reviews

## Abstract

With the increasing number of research priority setting (RPS) exercises, systematic reviews synthesising their findings have also grown in prevalence. While these reviews offer a structured way to compare methodologies, identify underrepresented stakeholder groups, and guide funding decisions, conventional systematic review methodologies, designed primarily for clinical and health research, often fail to capture the complexity, contextual nuance, and participatory nature of RPS. In this commentary, we critically examine these limitations and propose methodological adaptations to enhance the relevance and utility of systematic reviews of RPS. Beyond knowledge generation, we highlight the broader implications of RPS, including its role in stakeholder engagement, research funding allocation, and policy translation, as well as its impact on how these exercises are synthesised. By re‐evaluating how systematic reviews of RPS are conducted, we advocate for context‐sensitive methodologies that better reflect the dynamic and iterative nature of research priority setting.

A research priority setting exercise (RPS) is a collective activity that involves stakeholders in deciding how to allocate resources to a group of people, often incorporating different ways of processing and managing various sets of data, as well as wider engagement with participants. The goal is to decide how resources—whether financial or otherwise —might be allocated to specific research topics, questions, or projects. Sometimes these decisions are made with resources already in play, while other groups and stakeholders run the priority setting exercises hoping that entities with resources will adopt their priorities. There are occasions that the priority setting exercise is used as a knowledge acquisition and advocacy tool to understand the priorities of certain groups for example, patients or make them more visible. Overall the knowledge ecosystem has evolved where RPS is being recognised as a key tool to set priorities [1, 2]. Decisions on research priorities define what research questions get supported, however, important research questions stay unanswered for years that raises questions whether we need to rethink how we do research. This mismatch can result in research waste—where priorities either fail to address real‐world needs or duplicate previous studies unnecessarily [3, 4, 5].

Although there have been no formal studies, a quick search in PubMed reveals that in 2024, 57 papers were published in PubMed under the tags “research priority setting,” “research prioritisation,” or “research prioritisation.” Between 1994 and 2024 the highest number of research priority published was in 2022 a total of 80. The increasing conduct of RPS has meant that increasingly researchers are publishing systematic reviews of research priority setting (RPS) [6, 7, 8, 9, 10, 11, 12, 13, 14, 15, 16, 17, 18, 19, 20, 21, 22, 23]. These reviews have diverse objectives—some aim to understand the methodological approaches used and compare them, while others identify gaps, such as which stakeholder groups have been underrepresented in priority setting exercises or identifying priorities for resource allocation of a certain research group.

RPS can significantly influence resource allocation decisions, making them highly political processes. They do not just provide knowledge on what is ranked research priorities, these exercises foster stakeholder engagement; they also offer opportunities to engage with stakeholders, build partnerships and trust, and secure buy‐in from participants that extends beyond the exercise into the conduct of the research process itself and it subsequent translation in policy and practice. RPS exercises are underpinned by various methodologies, such as the Delphi method and the nominal group technique, each influencing the outcomes in distinct ways. For instance, the Delphi method's iterative nature may lead to more refined priorities, while the nominal group technique could promote immediate consensus among stakeholders.

In some systematic reviews, the topics that emerge from these priority setting exercises are explored. However, applying the conventional framework of systematic reviews in health research directly to RPS exercises is overly simplistic and may not adequately capture the complexity, contextuality and varying scope of RPS. For the purposes of a systematic review, RPS is typically treated as a knowledge‐acquisition exercise, as we use the outputs of all research priority‐setting activities as new results or evidence. There is only one exception that we will highlight later.

While RPS exercises are becoming increasingly prevalent, there is no consensus on the best way to synthesise them through systematic reviews. Many current approaches adapt methods from health research frameworks, which may not be suitable due to the inherently participatory nature of RPS exercises. In this paper, we rationalise for and discuss the need for tailored guidance for systematic reviews of RPS which can capture adequately the unique complexities it navigates.

The questions we need to ask are: why do we need to synthesise these exercises, and if so how can such synthesis adequately capture what the RPS process entails? For example, imagine a research group in the UK decides to run a research priority setting exercise involving patients and other stakeholders to support their decisions on which research projects are most likely to impact patient health. Now, let's imagine that this group primarily receives funding from UK‐based research funders and organisations. If they run a systematic review of RPS in their clinical focus and identify 10 priority setting exercises, but all 10 were conducted in countries with vastly different cultural and economic contexts from the UK, how could a summary of these exercises provide meaningful support to the UK‐based research group, which focuses on local contexts and funders? One could argue that the processes used in other contexts might offer insights into what could work or not work for the group.

In this paper, we suggest a few conceptual adjustments on how to conduct systematic reviews of RPS, to make them more relevant and useful for the groups that conduct them.

**Screening question:** One key question the group should ask is whether they need a systematic review of research priority setting exercises. If the group or organisation has a very specific scope that requires engagement with local stakeholders—and the buy‐in of those stakeholders is crucial for ensuring the success of the priority setting process, securing funding, and recruiting participants—then the group needs to consider whether the systematic review would add any value. It is helpful to check whether a similar review was recently conducted with a comparable group of patients, and they might find the results useful. However, this alone does not help build the partnership, trust, and buy‐in that the process requires. It is possible that the group could adopt the priorities from another group while finding alternative ways to build partnerships and trust.

## 1 Structure for the Research Question

The framework recommended as part of the REPRISE reporting checklist [24]. The REPRISE checklist suggested the GhePoR(T) framework for structuring the questions for research priority setting exercises.

– **G**eographical scope.
– **he**alth area, field, focus.
– **Po**pulation.
– **R**esearch Area and Question.
– **T**arget audience of the priorities (this is optional).

We would recommend a similar framework for systematic reviews of RPS. We realise on some cases, the framework might not have the flexibility to adjust when the focus is not a geographical area or clinical area so we have a suggested more concise framework as an alternative available ‐ PROFS framework.

- **P (Perspective):** What types of research priority setting are you focusing on? Perspective (P) refers to the type/stance of the RPS exercise (e.g., method‐defined such as Delphi/JLA; setting‐defined such as national funder or service‐user led; or purpose‐defined such as advocacy or implementation‐oriented).
- **R (Restrictions):** What are restrictions of the priority setting exercise? These are usually driven by the values and scope of the organisation conducting the priority setting, such as only focusing on priorities within the UK. This could be place, population or data or resources?
- **O (Outcome):** Are there any specific findings the review aims to uncover, such as mapping and scoping the priorities or specific factors that facilitate the engagement of individuals in the priority setting process, or identifying the most common priorities among certain stakeholder groups?
- **F (Focus):** Is the focus on the process of priority setting? Focus (F) refers to the analytic angle of the review (process, outcomes/priorities, evaluation/impact, or a combination)., the outcomes, evaluation of the success of the exercise or a combination of them?
- **S (Stakeholders/interest holders):** If stakeholders are involved, are there specific stakeholders you are focusing on?

For instance, in a systematic review focusing on mental health research priorities in the UK, the Population could be defined as stakeholders including patients, clinicians, and policymakers, with Restrictions being limited to studies conducted within the UK context.

## 2 Search Strategy

Currently, there is no validated search filter for identifying RPS studies in bibliographic databases. We suggest a few search strategies (Table 1) based on common terminology found in published RPS studies and our previous experiences conducting reviews in the field; however, further validation is required to ensure its accuracy and completeness. The elements of the PROFs or GhePoR(T) framework can help improve the specificity of searches. However, because the reporting of research priority‐setting exercises still needs improvement, we would advise exercising caution when conducting highly specific searches. Future research should focus on developing a standardised, high‐sensitivity filter for RPS studies.

**Table 1:** Suggested search strategies for systematic reviews of research priority setting.

Description	Search Strategy
Pubmed	1. “Health Priorities”[Mesh] AND research* 2. “Priority Setting” OR “Research Priorit*” OR “Setting Priorities” OR “Priorities in research” OR “Research Agenda” OR “Agenda setting” 3. 1 AND 2
Ovid (MEDLINE) (23)	research adj3 (priorit$ or agenda or strateg$). kw, ti
OVID (EMBASE) (24)	1. (research adj3 priorit$). tw. 2. (research adj3 agenda$). tw. 3. or/1–2

Regarding grey literature, this would be critical with systematic reviews on priority setting exercises. A reasonable number of the literature in the field is published on the website of organisations rather than peer reviewed publications.

The information specialist needs to consider that there is a construct between the published priority setting exercise and unpublished ones. For example‐ Funding organisations hold several panel sessions every year, and most countries have some form of a research organisation or department that allocates resources for research. Therefore, hundreds of prioritisation exercises happen each year. The details of these engagements often do not make it into published literature and sometimes not even into the funding reports (Nasser 2017, Nasser 2018). This is in contrast to studies that involve a new strategy to conduct priority setting exercises that more often get published in peer reviewed literature. We do not currently have a quantitative estimate of the difference to calculate the amount of publication bias that it creates. However, this can potentially lead to an imbalance in literature and consequently some level of a publication bias (meta‐bias). Therefore, we recommend that reviewers and information specialist consider this when searching additional resources or drawing conclusion from studies. One way to address the issue is conducting a survey for informal research priority setting exercises or an evaluation of websites of funding bodies or other organisations for reports.

## 3 Critical Appraisal of Studies

There are different types of aims and remit that systematic reviews of research priority setting have that can influence whether and how the reviewers appraise the studies. These types could include, amongst others:

- Systematic review of priority setting, and focus is on quality of reporting of the priority setting
- Systematic reviews of priority setting and focus on the quality of implementation of certain steps of the priority setting exercise e.g. the quality stakeholder engagement or the ethical integration in the process
- Systematic review of studies that evaluate priority setting exercise ‐ then you have two levels of potential quality assessment depending on what's your focus ‐ the quality of the design of the evaluation and the quality of the conduct of priority setting‐ examples
- Systematic review of research priority setting focusing on the priorities only ‐ then the quality issue is the level of clarity around the details of the priorities and the underlying nuances or narratives integrated with it and the detail and quality of the methods around translation the discussion into the question [25].

The reviewer should first differentiate whether they are appraising the studies that contributed to the design or evaluation of priority setting exercise or looking at the quality of the priority setting exercise itself. The latter is a complicated issue as the “quality assessment” implies that there is a concept what is a good priority setting and what is not, however, there is currently not enough studies to actually judge that so usually the evaluation is focused on issues like quality of reporting that you could do for example with the REPRISE tool [24]. There are elements in the priority setting exercise that we can evaluate their quality like for example the stakeholder engagement process, the economic evaluation underneath the priorities setting or the quality of evidence reviews that contributed to it.

If the systematic review focuses on the priorities or outcomes of research priority setting, we could assess the clarity of the priorities based on their ability to be accepted by researchers or implemented. For example, consider the two priorities below – one from Armstrong (2020) [26], which involved individuals with dementia and their carers, and one from a James Lind Alliance's Priority Setting Partnership with affected individuals, their carers, and service providers [27]. The one from Bethel (2018) demonstrates much more clarity and focus for researchers to follow. In contrast, the one from Armstrong is vague and general, allowing researchers to adapt the question in ways that may deviate significantly from what the stakeholders involved in the process initially intended or hoped for [27].

Priority 1 (Need for therapist to prevent, cure or slow Dementia with Lewdy bodies)

Priority 2 (from Bethel 2018) “*Among persons with dementia, what are the effects of non‐pharmacological treatments compared to pharmacological treatments on behavioural and psychological symptoms of dementia? Can non‐pharmacological treatments replace, reduce or be used in conjunction with pharmacological treatments for managing behavioural and psychological symptoms of dementia?* [27]”

Our suggested approach is that you rank the topics based on level of clarity. Clarity refers to the details of the question itself (similar to a structure research question like PICO, PECO or others that is used in systematic reviews or research) along with the context (the rational, the lived experience or the underlying data that resulted the stakeholder choose the research question and prioritise it). Example of a high level of clarity priority is priority 2 example that provides details on the direction of research that the stakeholders intend to go, medium level of clarity is priority 1 that gives some details but provides a lot of room and interpretation. We would have a third level of low level of clarity that would apply for priority setting that general would say dementia research is a priority.

## 4 Synthesis of the Studies

There are two approaches to synthesising RPS exercises [1]: synthesising evaluation studies of RPS exercises, or [2] synthesising the characteristics and outcomes of RPS themselves.

For the former, depending on the nature of the evaluation, the synthesis may be qualitative or quantitative and will depend on which outcomes are being measured and analysed. For example, if you are looking at the impact of research priority setting with regard to identifying more research projects that can impact health inequalities. You will look at priority setting exercises that have long term evaluation attached to them and look at what priority setting exercise with what characteristic is more likely to result in studies that have an impact. In this speculative scenario, you might for example find out that involving stakeholders in meetings is more likely than the survey.

However, we currently cannot do these types of systematic reviews, as there are not enough studies focusing on this area.

Most currently evaluation studies focus on the process of the priority setting [28, 29].

The other type of synthesis focuses mainly to understand the characteristics of priority setting or understand the nature of the identified priorities itself. These two other types of reviews are as follow:

– **Focus on the “process” of the priority setting exercise:** This would partially depend on the focus of the findings. For example, if the focus is on understanding what facilitates or creates barriers in stakeholder engagement during the priority setting process, well‐recognized qualitative evidence synthesis approaches or realist review approaches might be used. However, if the question focuses on why and how certain stakeholders agree to participate in the process (or not), a different approach, such as a realist review, would be more appropriate.
– **Focus on the “priorities” at the end of a research priority setting exercise:** Current systematic reviews typically take a simplified approach that involves outlining counting priorities or trying to find mutual themes across them. We propose an alternative method, termed “meta‐priorities synthesis.” The approach begins by identifying primary l themes that encompass several priorities but tagged with their characteristics. Then, a second level of themes would be identified that cover multiple first‐level themes, and so on, until you reach a top level of priorities. Any themes beyond the third level are considered meta‐priorities.

The nature of the meta‐priorities depends on the sources of the priority‐setting exercise. For example, if you have a series of research priority setting exercises conducted in Brazil, Germany, and China, and the meta‐priority is mental health, this would be considered a cross‐country meta‐priority. If the series of research priority setting exercises focuses on different stakeholder groups ‐ some engaging only with patients, others with clinicians ‐ the meta‐priorities would be cross‐stakeholder meta‐priorities. We suggest that authors repeat the thematic analysis for different cross‐groups until they find a series of meta‐priorities across different “characteristics” that provide them an overview how priorities are expressed in different clusters and where there are similar value judgements made. This then can become a useful tool for policy makers to make decisions on priorities building activities of smaller or regional organisations or making global decisions based on national or regional priorities. The grouping of themes and the selection of meta‐priorities will depend on the structure of the question as outlined before. When meta‐priorities are identified, it is important to interpret them in conjunction with the available information on context and value judgements.

In both of these synthesis approaches, we considered research priority setting as a knowledge acquisition exercise, where the priorities are the results and new knowledge is created. However, there is one instance where a different approach to synthesis is appropriate. If the focus of the review is to understand the historical development of research priorities in a certain field or community and its implications for research infrastructure, we recommend using established methods from the historical sciences to analyse or synthesise these data. In these cases, all included research priority‐setting exercises are treated as political interactions, explored in the context of how they have influenced and shifted the allocation of resources and affected the research agenda.

## 5 Conclusion

Systematic reviews of RPS must evolve beyond traditional evidence synthesis methods to fully capture the participatory and contextual nature of these exercises. We propose the PROFS framework as a step toward refining these reviews. Future research should focus on [1]: validating search filters for RPS studies [2], refining quality assessment criteria, and [3] testing the PROFS framework in real‐world systematic reviews. By adapting our approach, we can ensure that systematic reviews of RPS provide meaningful insights that support better research agenda‐setting and funding decisions. Systematic review of RPS can be used to inform agenda setting when organisations cannot afford to conduct a full research priority‐setting exercise, provide insights into what has and has not worked previously to support the design of better exercises in the future, or help define the scope of future work by identifying gaps in knowledge rather than duplicating existing priorities.

## Author Contributions

**Mona Nasser:** conceptualization, methodology, investigation, data curation, project administration, writing – review and editing, writing – original draft, formal analysis. **Sumanth Kumbargere Nagraj:** writing – review and editing, methodology. **Seilin Uhm:** writing – review and editing, methodology. **Prashanti Eachempati:** methodology, writing – review and editing. **Soumyadeep Bhaumik:** methodology, writing – review and editing.

## Funding

The authors have nothing to report.

## Conflicts of Interest

The authors declare no conflicts of interest.

## Data Availability

Data sharing not applicable to this article as no datasets were generated or analysed during the current study.
